# Positive Impact of Pulsed Electric Field on Lactic Acid Removal, Demineralization and Membrane Scaling during Acid Whey Electrodialysis

**DOI:** 10.3390/ijms20040797

**Published:** 2019-02-13

**Authors:** Guillaume Dufton, Sergey Mikhaylin, Sami Gaaloul, Laurent Bazinet

**Affiliations:** 1 Institute of Nutrition and Functional Foods (INAF), Dairy Research Center (STELA) and Department of Food Sciences, Université Laval, Québec, QC G1V 0A6, Canada; guillaume.dufton.1@ulaval.ca (G.D.); Sergey.Mikhaylin@fsaa.ulaval.ca (S.M.); 2 Laboratoire de Transformation Alimentaire et Procédés ÉlectroMembranaires (LTAPEM, Laboratory of Food Processing and ElectroMembrane Processes), Université Laval, Québec, QC G1V 0A6, Canada; 3 Parmalat, Victoriaville, Québec, QC G6P 9V7, Canada; sami_gaaloul@parmalat.ca

**Keywords:** acid whey, electrodialysis, pulsed electric field, demineralization, scaling, lactic acid removal

## Abstract

The drying of acid whey is hindered by its high mineral and organic acid contents, and their removal is performed industrially through expensive and environmentally impacting serial processes. Previous works demonstrated the ability to remove these elements by electrodialysis alone but with a major concern—membrane scaling. In this study, two conditions of pulsed electric field (PEF) were tested and compared to conventional DC current condition to evaluate the potential of PEF to mitigate membrane scaling and to affect lactic acid and salt removals. The application of a PEF 25 s/25 s pulse/pause combination at an initial under-limiting current density allowed for decreasing the amount of scaling, the final system electrical resistance by 32%, and the relative energy consumption up to 33%. The use of pulsed current also enabled better lactic acid removal than the DC condition by 10% and 16% for PEF 50 s/10 s and 25 s/25 s, respectively. These results would be due to two mechanisms: (1) the mitigation of concentration polarization phenomenon and (2) the rinsing of the membranes during the pause periods. To the best of our knowledge, this was the first time that PEF current conditions were used on acid whey to both demineralize and deacidify it.

## 1. Introduction

Acid whey is the principal co-product of a wide variety of dairy products, such as fresh cheese, caseinate, and Greek yogurt. Its production is increasing every year to answer the actual popularity rise of aforementioned products, and its valorization represents a great deal for dairy industries [1,2]. However, most acid whey applications need a preemptive drying for volume, transport, and preservation enhancement, but acid whey processing is hindered by its high hygroscopic character resulting in major operational problems during its drying. Saffari & Langrish in 2014 and Chandrapala & Vasiljevic in 2017 explained the inability to produce good quality dried powders from acid whey as being due to its high lactic acid and mineral contents. Both lactic acid and mineral contents are responsible for a decrease in glass transition temperature and, consequently, to lower crystallization yields [3,4]. To allow acid whey subsequent valorization, the dairy industry used several processes, such as combinations of electrodialysis (ED), ion-exchange resins, and nanofiltration [5,6] to remove salts and lactic acid. Nevertheless, the cost and environmental impact of the above-mentioned processes are tremendous and mostly attributed to the use of ion-exchange resin [7]. Hence, in the recent years, a number of studies were published regarding acid whey processing by optimizing either nanofiltration [8,9] or ED processes [10,11], trying to provide better cost-effective and eco-efficient alternatives. So far, ED processes allowed for reaching suitable deacidification (44%) and demineralization (67%) rates that have been reported as sufficient for the acid whey to be spray-dried while obtaining powders of acceptable quality [9]. However, whatever the configuration or parameters used, these ED processes were subject to major scaling (mineral fouling) on membrane surfaces [11], making the transposition of ED alone, or without pretreatments, impossible at an industrial scale.

Membrane fouling and scaling during ED have been studied by several authors, and the major scaling agents reported and found in acid whey were calcium and magnesium [12,13,14]. To mitigate or even sometimes completely suppress scaling consisting of these minerals, a particularly effective method was recently reported in the literature: the use of pulsed electric fields (PEF). Cifuentes et al. (2011), Mikhaylin et al. (2014), and Andreeva et al. (2018) successfully demonstrated the favorable impact of using PEF on scaling mitigation during ED of model salt solutions [15,16,17]. Moreover, the application of PEF during ED was also reported to enhance ion migration thanks to a mitigation of the concentration polarization phenomenon [18,19,20]. In all previous studies of ED under PEF, demineralization efficiency and membrane scaling were the main issues. However, deacidification of real acid food solutions in conventional ED under PEF has never been reported in the literature.

In this context, the goal of the present study is to demonstrate the efficiency of applying PEF during ED for simultaneous deacidification and demineralization of acid whey. The specific objectives were, therefore, (1) to evaluate the impact of PEF on the deacidification and demineralization rates of acid whey, (2) to test the effect of different pulse/pause combinations on demineralization and deacidification rates, and (3) to ascertain the potential effect of PEF on membrane scaling mitigation.

## 2. Results and Discussion

### 2.1. Whey and Recovery Solutions Analysis

#### 2.1.1. Lactate Migration

**Figure 1:**
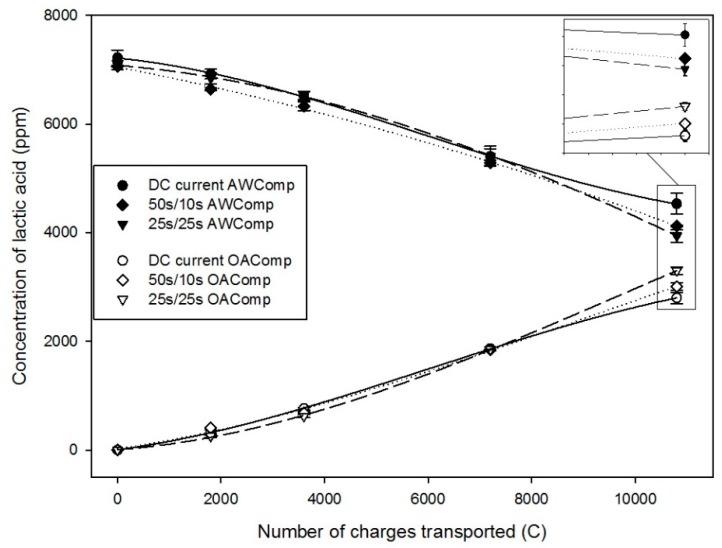
Evolution of lactic acid (in ppm) in the AWComp (black points) and OAComp (white points) for the DC (dots), pulsed electric field (PEF) 50 s/10 s (rhombuses), and PEF 25 s/25 s (inverted triangles) current conditions.

Regarding lactate migration, the application of the two pulse/pause combinations had a significant impact, in comparison with the DC current control condition, on the deacidification rates obtained (P < 0.01). Indeed, in the DC current condition, the lactate concentration decreased in the AWComp from 7.22 ± 0.14 g/L to 4.53 ± 0.19 g/L (P < 0.001), corresponding to a 37.2% ± 1.7% deacidification rate (Figure 1). In parallel, the corresponding acidification rate observed in the OAComp, with a recovery of 2.80 ± 0.10 g/L of lactate, was 38.8% ± 1.5% of the initial lactate concentration. For the PEF 50 s/10 s condition, the lactate concentration in the AWComp dropped from 7.07 ± 0.02 g/L to 4.13 ± 0.01 g/L (P < 0.001), corresponding to a deacidification rate of 41.6% ± 0.1%. In the OAComp, the final lactate concentration reached 3.01 ± 0.07 g/L which represented 42.6% ± 1.0% of the initial lactate concentration. Finally, for the PEF 25 s/25 s condition, lactate concentration in the AWComp decreased from 7.09 ± 0.08 g/L to 3.94 ± 0.11 g/L (P < 0.001) corresponding to a deacidification rate of 44.4% ± 1.1%. In parallel, the final lactate concentration in the OAComp reached 3.30 ± 0.07 g/L and an acidification rate of 46.6% ± 1.5% of the initial lactate concentration. The final deacidification rates obtained were in direct correlation with the final lactate concentration in the OAComp: DC current < PEF 50 s/10 s < PEF 25 s/25 s.

As expected, lactate migrated through the AEMs from the AWComp to the OAComp during the three effective hours of current application, equivalent to a number of charges transported of 10800 C. For all conditions, the lactate recovery was similar to the deacidification observed, meaning no lactate was trapped or involved in any fouling and/or scaling reaction [11]. The application of PEF was demonstrated to be efficient for the improvement of the deacidification rates: the 50 s/10 s pulse/pause condition allowed an increase of 10%, while the 25 s/25 s condition allowed an improvement of 16%. The effect of PEF on the migration rates has already been demonstrated on salt solutions by several authors [15,17,18,19] but, in the present study, it was demonstrated for the first time on lactate in acid whey. According to these authors, PEF has a favorable impact on the reduction of concentration polarization at the membrane vicinity and, thus, on the reduction of water splitting phenomenon. Indeed, by applying pulsed current, the pause lapses allow the ion’s concentrations at the membrane’s boundary layers to return to a value close to the bulk solution concentration. By doing so, when the current is applied again, the migration efficiency can momentarily exceed the maximum imposed by the limiting current density, thanks to a lower ohmic resistance and a better ion availability [20]. The higher migration rate obtained for the PEF 25 s/25 s condition confirmed the results reported previously by Cifuentes-Araya et al. (2011) [15] for the ratio *r* = 1 (10 s/10 s) on salt solutions. Pelletier et al. (2015) [21] also obtained their best deacidification conditions for pulse/pause combinations of ratio *r* = 1 (1 s/1 s and 2 s/2 s) on cranberry juice. Their experiments resulted in a 12 to 19% improvement of their migration rates in PEF modes in comparison to DC one, depending on the organic acid anion, for a longer run (4 effective hours) but at lower current density than in the present study.

#### 2.1.2. Protein Content

Before and after ED, the total protein content was determined to evaluate whether whey proteins were transferred or lost during the process. The results are shown in Table 1, and allowed us to conclude that the whole protein content was preserved.

#### 2.1.3. pH

**Figure 2:**
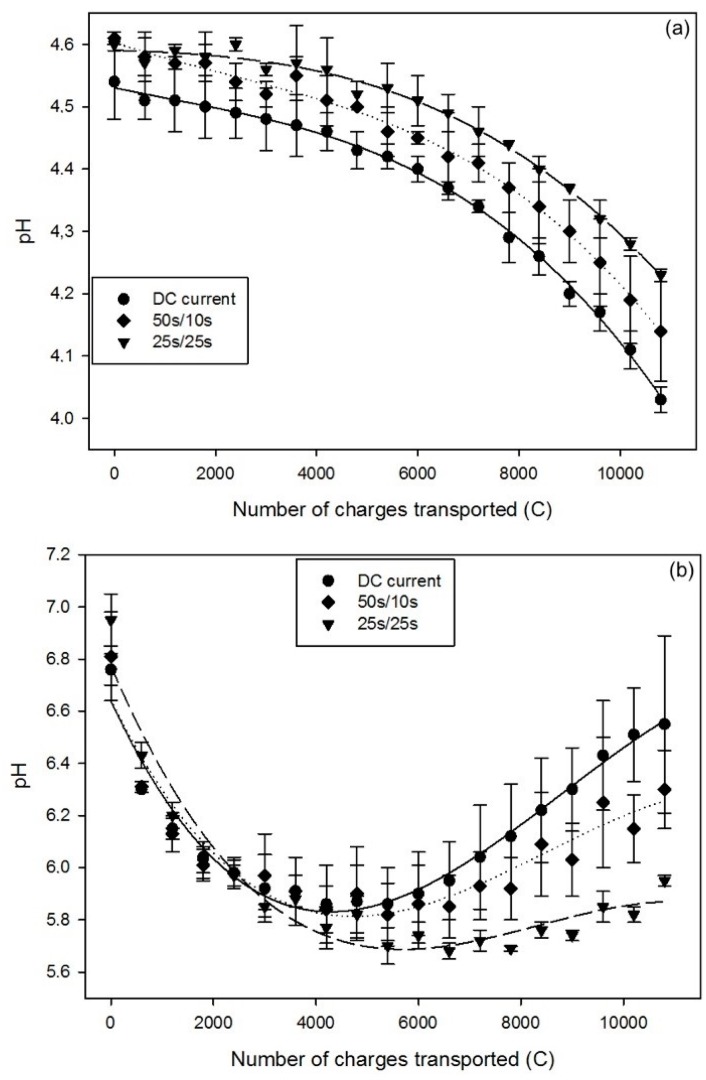
pH evolution for DC (dots), PEF 50 s/10 s (rhombuses), and PEF 25 s/25 s (inverted triangles) current conditions in (**a**) AWComp and (**b**) OAComp.

The pH of whey, in the AWComp, significantly decreased for all conditions after the three effective hours of current application, or number of charges transported of 10800 C (P < 0.01). As shown in Figure 2a, the pH decreased from an averaged initial value of 4.58 ± 0.03 to final values of 4.03 ± 0.02 for DC current, 4.14 ± 0.08 for PEF 50 s/10 s, and 4.23 ± 0.01 for PEF 25 s/25 s. Due to higher variability during PEF 50 s/10 s condition, no significant difference was observed between this condition and both other (P = 0.74 and P = 0.11 with DC, and PEF 25 s/25 s current conditions, respectively). However, the pH variation between the beginning and the end of the treatment for DC and PEF 25 s/25 s current conditions was significantly different (P = 0.04). For all three conditions, the pH evolution in the AWComp presents similar profiles. They all present one inflection point at around 7200 C for the DC and PEF 50 s/10 s current conditions, and at around 6600 C for PEF 25 s/25 s. From the initial pH value, the application of either DC, PEF 50 s/10 s, or PEF 25 s/25 s current resulted, at the end of the process, in a pH decrease of 11%, 9%, and 7%, respectively. Regarding OAComp (Figure 2b), the pH evolution had a similar trend during the treatment for all conditions: an initial decrease to reach a minimum value followed by a subsequent increase (P < 0.05). However, the subsequent increase appeared at a different number of charges transported depending on the current condition. For the DC current condition, pH decreased from 6.84 ± 0.11 to a minimum of 5.86 ± 0.15 at 4200 C, and then increased to reach a final pH of 6.55 ± 0.34. For PEF 50 s/10 s and 25 s/25 s the pH reached respective minima of 5.82 ± 0.12 at 5400 C and 5.68 ± 0.03 at 6600 C, and then rose, to final respective pH values of 6.30 ± 0.15 and 5.95 ± 0.02 (Figure 2b). As for the pH evolution in AWComp, the values of the final pH in OAComp were statistically similar between the PEF 50 s/10 s and the two others current conditions (P = 0.39 and P = 0.18 with DC and PEF 25 s/25 s current conditions, respectively) while the PEF 25 s/25 s and DC current conditions were significantly different (P = 0.03).

pH decreases in the AWComp during acid whey deacidification were reported, in previous studies in DC current conditions [10,11], to be caused by the dissociation of lactic acid following lactate migration, and by protons generated from water splitting phenomenon appearing mainly, after a certain time, on the AEM diluate side, as observed by other authors on model salt solutions [22,23]. However, the results of the current study demonstrate that the application of an adjusted PEF ratio was efficient in the reduction of such pH variations. As already mentioned, PEF has been reported to have a favorable impact on the reduction of the water splitting phenomenon during salt solution electrodialysis. This effect is described by Sistat et al. (2015) [20] and Malek et al. (2013) [19] as the restoration of the ionic concentrations at the membrane boundary layers during the pause periods, hence limiting the concentration polarization. The higher final pH value in AWComp for the PEF 25 s/25 s current condition (Figure 2a), in comparison to the DC one, was probably caused by the lower production of protons in the whey through the diminution of water splitting phenomenon at the AEM’s interface, in contact with the acid whey. Regarding OAComp (Figure 2b), the evolution of pH has been described in a previous study [11] for the DC current condition. The initial pH decrease in all conditions was likely caused by the migration of protons from the AWComp through the CEMs, followed by other slower anions, such as phosphates or organic acids, increasing the buffer capacity of the solution, hence, the pH stabilization after 4200 C. After 7200 C, the pH rose again due to the aforementioned water splitting phenomenon occurring after further demineralization of the AWComp. However, regarding the results of the PEF 25 s/25 s current condition, the decreasing phase lasted longer and remained constant while increasing at a drastically slower pace than in the DC current condition. This is further proof of the favorable impact of applying PEF on the water splitting phenomenon reduction.

#### 2.1.4. Conductivity

**Figure 3:**
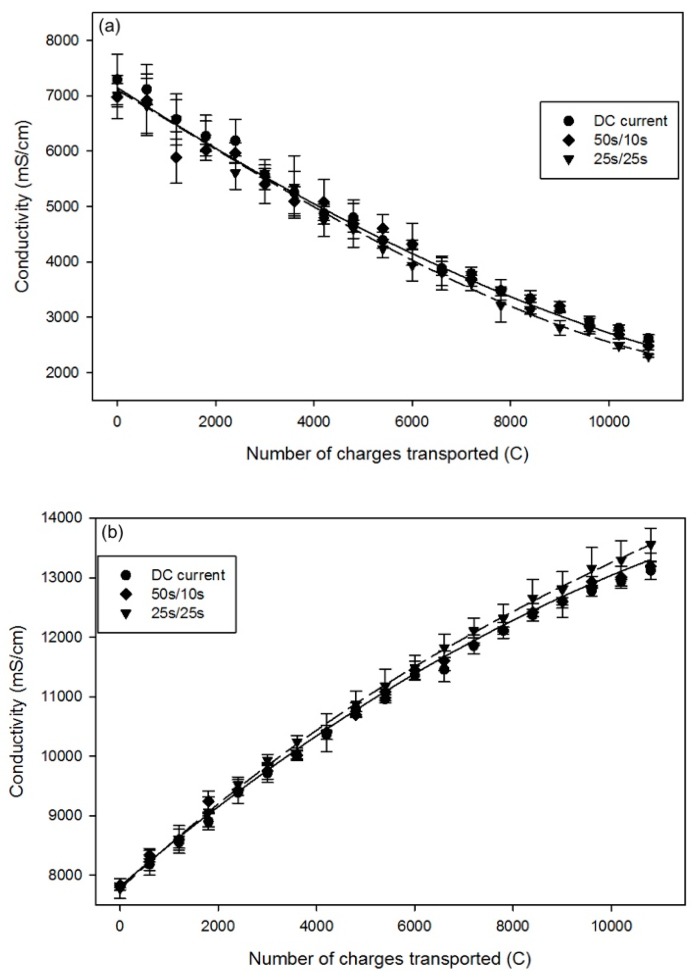
Conductivity evolution for DC (dots), PEF 50 s/10 s (rhombuses) and PEF 25 s/25 s (inverted triangles) current conditions in (**a**) AWComp and (**b**) OAComp.

In the AWComp solution, the conductivity decreased significantly during the 3-hour effective treatment for all three conditions (P < 0.003). It dropped from an averaged initial value of 7.09 ± 0.35 mS/cm to final values of 2.62 ± 0.06 mS/cm for the DC current condition, 2.49 ± 0.08 mS/cm for PEF 50 s/10 s, and 2.30 ± 0.03 mS/cm for PEF 25 s/25 s (Figure 3a). The demineralization rate of the DC current and PEF 50 s/10 s current conditions were very similar: 64.0% ± 2.5% and 64.2% ± 3.0% respectively (P = 0.09), while the PEF 25 s/25 s condition showed a statistically higher demineralization rate of 67.1% ± 0.7% (P < 0.02 with both DC and PEF 50 s/10 s current conditions). Regarding OAComp, the conductivity also increased in a similar way regardless of the current condition applied, from an initial averaged value of 7.81 ± 0.09 to 13.12 ± 0.15, 13.19 ± 0.23, and 13.56 ± 0.28 mS/cm for the DC, PEF 50 s/10 s, and PEF 25 s/25 s current conditions respectively (*p* < 0.001) (Figure 3b). Here, again, there was no significant difference between the DC and PEF 50 s/10 s current conditions (P = 0.92) with mineralization rates of 40.5% ± 0.3% and 40.6% ± 0.9% respectively, but the PEF 25 s/25 s current condition showed a better rate of 42.7% ± 0.1% (P < 0.02 and P < 0.03 with DC and PEF 50 s/10 s current conditions respectively).

The AWComp demineralization and OAComp mineralization were directly correlated to the ions’ migration from AWComp to OAComp through the membranes. The better demineralization/mineralization rates for PEF 25 s/25 s current condition confirmed the previous results on lactate migration and the hypothesis of migration enhancement thanks to an appropriate PEF condition. However, as reported by Lin Teng Shee et al. (2008) [24] regarding solution demineralization using bipolar membranes, the H^+^ and OH^−^ produced during the process contribute more to the conductivity than other ions. Water splitting phenomenon occurring during the experiments in our study, producing such ions, might thus have impacted the conductivity measurements and not reflect the real migration of mineral ions by overestimating the final mineral concentration.

### 2.2. Global System Resistance and Relative Energy Consumption

**Figure 4:**
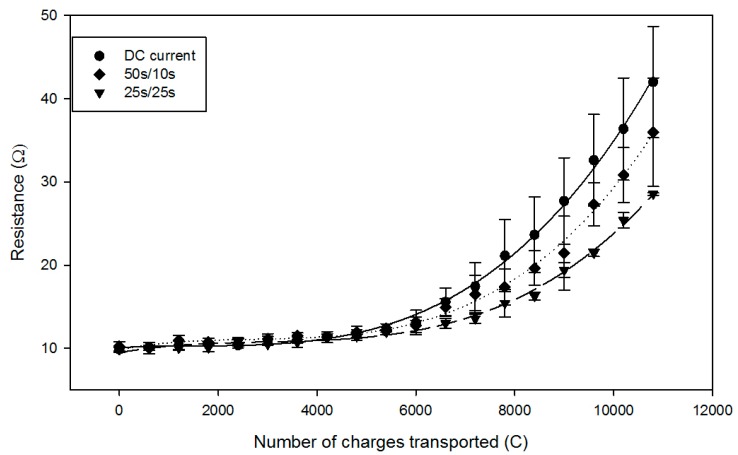
Global system resistance evolution for DC (dots), PEF 50 s/10 s (rhombuses), and PEF 25 s/25 s (inverted triangles) current conditions.

The global system resistance was significantly affected by the application of PEF. It increased from an initial value of 10.0 ± 0.4 Ω to values of 42.0 ± 6.7 Ω (increase of 4.2-folds), 36.0 ± 6.5 Ω (3.6-times increase), and 28.6 ± 0.2 Ω (2.8-folds increase) for the application of DC current, PEF 50 s/10 s, and PEF 25 s/25 s, respectively (Figure 4). Due to high values of standard deviation for DC and PEF 50 s/10 s current conditions, no significant difference was observed between them (P = 0.31). However, the PEF 25 s/25 s current condition showed a significantly lower resistance value in comparison with the DC current condition (P = 0.024). Moreover, depending on the applied current condition, the increase in global resistance appeared at a different number of charges during the process, with PEF conditions delaying the appearance of the resistance increase: around 6000 C for the DC current condition, 6600 C for the PEF 50 s/10 s condition, and 7200 C for the PEF 25 s/25 s condition. Regarding relative energy consumption, only power supply consumption was taken into account as the pumps’ energy consumption represents a negligible part of the global consumption, even if the process time is doubled. The DC current condition consumed 9.33 ± 1.38 Wh/g of lactic acid recovered, while the PEF 50 s/10 s and PEF 25 s/25 s consumed 7.88 ± 0.64 and 6.21 ± 0.30 Wh/g of lactic acid recovered, respectively: this corresponds to a 33% less relative energy consumption for the 25 s/25 s condition. Here, again, only the PEF 25 s/25 s was significantly different from the DC current condition (P = 0.03).

These different values in number of charges transported, where the global resistance increased during the process, can be related to the inflection points previously observed for the pH, and would correspond to the formation of a significant scaling/fouling on the membranes [11], resulting in further water dissociation. In the present study, the application of PEF would have slowed down the appearance of scaling/fouling and, consequently, of water splitting, as demonstrated by Cifuentes-Araya (2013) [25] and Andreeva et al. (2018) [16] in studies on the same model salt solutions enriched with scale-forming ions (Ca^2+^, Mg^2+^, …). Such a delay in scaling formation allowed, consequently, a decrease in the final global system resistance of 32% for PEF 25 s/25 s condition in comparison with DC current but, also, a significant saving in relative energy consumption of around 33%.

### 2.3. Membrane Analysis

#### 2.3.1. Membrane Photographs

**Figure 5:**
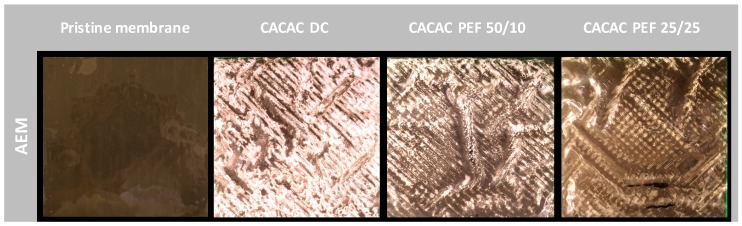
Entire anion-exchange membranes’ (AEMs’) anode sides (10 cm^2^) photographs before and after ED for all three current conditions. AEM comprised of A1 and A2 with no visible differences.

To evaluate whether there is formation of scaling/fouling on membranes, after each ED run and after rinsing the cell during 5 min with water, the stack was dismantled, and photographs of each membrane were taken (Figure 5). For all three current conditions, the AWComp side of the AEMs were all free of any visible fouling or scaling. However, on the OAComp side of the AEMs, a white plaster-like scaling was visible for the three conditions, with decreasing quantity from DC to PEF 50 s/10 s and then to PEF 25 s/25 s current conditions. Regarding the CEMs, no scaling or fouling was observed on both sides of the membranes, as previously reported [11].

As in the previous study by Dufton et al. [11], the scaling on the AEMs affected the pH variations, and the global system resistance increased during the process. The mitigation of this scaling by application of PEF was visible through the apparent reduction of scaling for the PEF current conditions: the scaling was more important after DC current application, slightly decreased by 50 s/10 s PEF application, and drastically decreased after the treatment with PEF 25 s/25 s.

#### 2.3.2. Membrane Thickness and Conductivity

**Figure 6:**
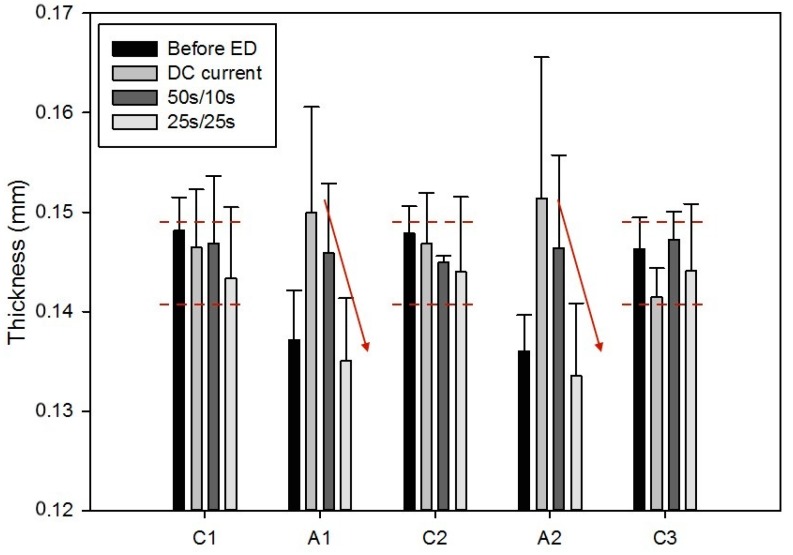
Membrane thickness measurements for all membranes of the ED configuration, before and after treatment, for the three different current conditions. The arrows show the thickness decreasing tendency depending on the applied current condition.

For all conditions, there was no significant difference between treatments for the AEM conductivity. However, the CEMs were similarly affected for all conditions in comparison to the pristine membranes: a decrease in conductivity of around 10% was observed for C1 (P < 0.02), while a 30% decrease was visible on the C2 and C3 membranes (P < 0.006). Regarding membrane thickness, as shown in Figure 6, the CEMs remained similar before and after ED treatment for all three current conditions (P = 0.119). The measurements on the AEMs had high variability caused by the irregular scaling on their surfaces, and no statistical difference was observed between the different conditions, but a clear tendency can be seen on the thickness means. The PEF 25 s/25 s AEM’s thickness after ED treatment is the one closest to the pristine membrane value.

Membrane conductivity followed the same pattern as in the previous study with DC current [11], regardless of the current condition. The decrease in conductivity for the CEMs would be due to the presence in the membrane of counterions having lower electrophoretic mobilities compared to initial Na^+^ counterions. Indeed, calcium and magnesium ions present in the acid whey and migrating through the CEM have lower electrophoretic mobilities (Ca^2+^: 1.07 × 10^9^ cm^2^/V∙s, Mg^2+^: 0.91 × 10^9^ cm^2^/V∙s) in comparison to sodium ions present in the pristine CEM (Na: 4.39 × 10^9^ cm^2^/V∙s) [26]. Despite the visible deposit on the AEMs, no impact was observed on conductivity, which means no significant damage was done to the membrane integrity. Regarding membrane thickness, the values for the CEMs showed that, regardless of the current condition, the ED treatment had no effect on the membranes. On the other hand, the thickness measurements for the AEMs illustrate, distinctly, the favorable effect of the application of pulsed electric field and especially of an optimized pulse/pause combination on deposit reduction.

### 2.4. Scaling Characterization

#### 2.4.1. Mineral Content

**Figure 7:**
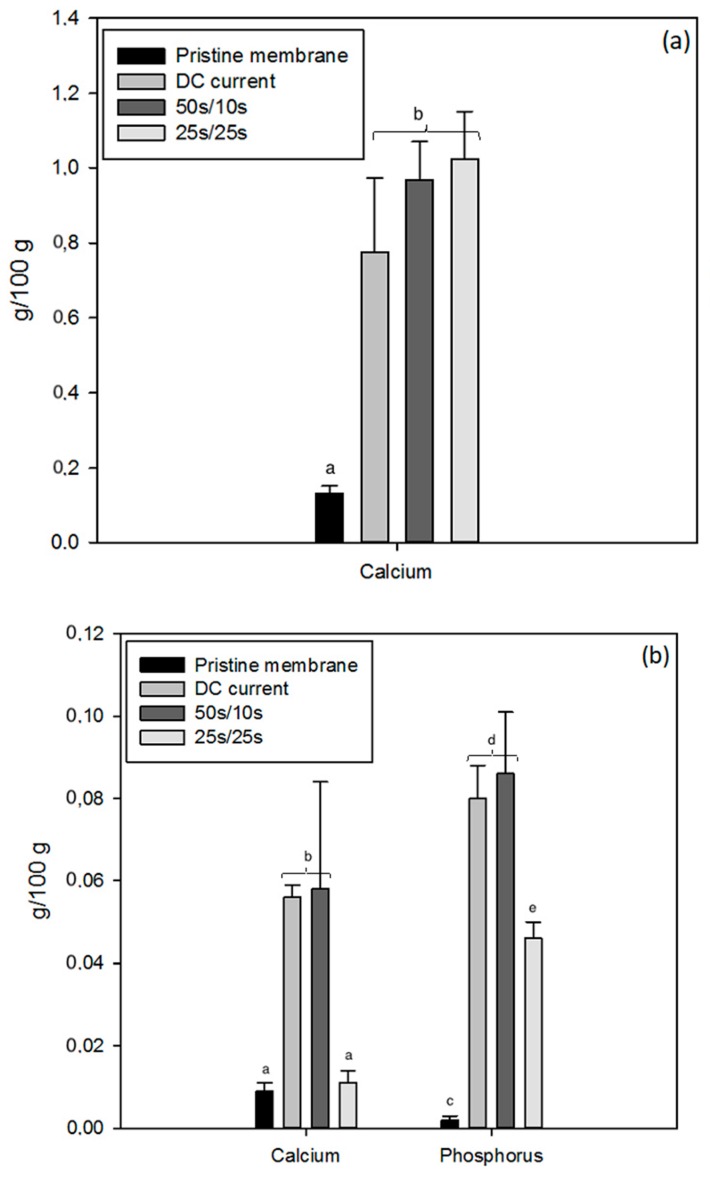
Calcium content of cation-exchange membranes (CEMs) (**a**) and calcium and phosphorus content of AEMs (**b**) for all three current conditions, in comparison with a pristine membrane. Different letters on histograms for each element means that there is a statistical difference between their values (P < 0.05).

The mineral composition of IEMs provided information concerning the deposit nature. All membranes were similar in content for potassium (0.05 ± 0.01 and 0.01 ± 0.00 g/100 g of CEM and AEM, respectively), sodium (4.72 ± 0.23 and 0.04 ± 0.01 g/100 g of CEM and AEM, respectively) and magnesium (0.02 ± 0.00 and 0.00 ± 0.00 g/100 g of CEM and AEM, respectively) for the three different current conditions. Nevertheless, the CEMs’ calcium amount after ED treatment increased around 5-fold in comparison with a pristine membrane (Figure 7a) (P < 0.001), and the application of PEF had no significant impact on this concentration (P > 0.16). However, the trend suggests that calcium concentration in the CEM was increased by PEF application. Regarding the AEMs, as shown in Figure 7b, calcium content increased significantly about 6-fold for the DC and PEF 50 s/10 s current conditions (P = 0.013) while remaining close to the initial value for the PEF 25 s/25 s current condition (P = 0.99). There were also differences in phosphorus content in the AEMs before and after processing treatment: it increased about 26 times for the PEF 25 s/25 s current condition while increasing 40-fold for the DC and PEF 50 s/10 s current conditions, in comparison with the pristine membrane (P < 0.01).

Once again, as already mentioned, the presence of calcium in the CEMs would be due to the residual ions inside the membrane’s nanochannels due to the late and slow migration of calcium during the process [26]. Regarding the AEMs, as reported in the previous study [11], the scaling observed on their surface seems to be mainly composed of calcium phosphate. Indeed, its precipitation reaction is triggered by alkaline conditions and implied, therefore, the occurrence of water splitting to locally obtain alkaline conditions on the AEM’s surface [27,28]. However, as shown in Figure 7b, the application of PEF with a pulse/pause combination of 25 s/25 s allows for significant reduction of the scaling amount. This effect was reported by other authors on saline solutions [15,16,17] but this is the first time that the application of an adjusted PEF is demonstrated to be efficient on scaling reduction during acid whey ED.

#### 2.4.2. Scanning Electron Microscopy (SEM), Energy Dispersive Spectroscopy (EDS), and X-ray Diffraction (XRD) Analyses

**Figure 8:**
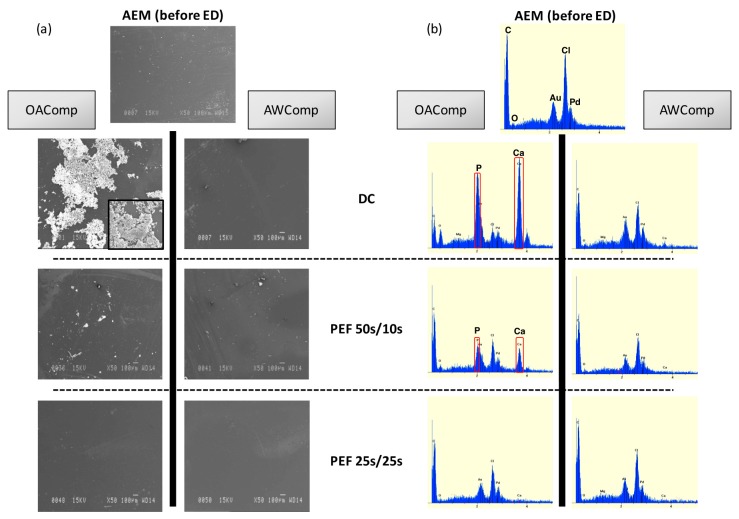
Scanning electron microscopy images with magnifications of 50× and 750× (**a**) and energy dispersive X-ray spectroscopy (**b**) of AEM sides for DC, PEF 50 s/10 s, and PEF 25 s/25 s current conditions compared to pristine membranes.

The CEMs’ observation by SEM showed an absence of deposit for all current conditions and membrane’s sides, with pictures similar to the pristine membrane. XRD analysis did not reveal any form of crystalline precipitate (Supplementary Data A) but very low amounts of calcium, similar for all conditions of current applied, which were visible in the elemental analysis by EDS. Regarding the AEM photographs obtained by SEM, the membranes’ AWComp side appearances were similar to the pristine membrane for all current conditions, while the membranes’ OAComp side from the DC current condition showed massive deposits (Figure 8a). This scaling can also be observed, to a lesser extent, on the AEMs’ OAComp side for the PEF 50 s/10 s condition, but is absent from the membranes from the PEF 25 s/25 s condition. The analysis of these membranes by XRD did not reveal any crystalline deposit, but the EDS showed high concentrations of calcium and phosphorus on the OAComp’ side for the DC current condition membranes and were relatively lower for the PEF 50 s/10 s current condition (Figure 8b).

Just as reported in the previous study [11], the CEMs were free from any form of fouling or scaling, and the traces of calcium observed were most likely due to the presence of free ions migrating into the membrane nanochannels. As for the AEMs, the major scaling visible on the DC current condition membranes’ OAComp side is similar to the one described in a previous study [11] and is known as an amorphous form of calcium phosphate (ACP: Ca_x_H_y_[PO_4_]_z_ nH_2_O, *n* = 3–4.5; 15%–20% H_2_O) [27,29]. Regarding the PEF 50 s/10 s current condition, the AEMs’ OAComp side was also subject to similar scaling but at a relatively lower level (around 3 times), while the PEF 25 s/25 s current condition AEMs were completely free of apparent scaling. It was necessary to moderate these SEM observations since the scaling for PEF 25 s/25 s current condition was extremely uneven (Figure 5) and already in lesser quantity than other conditions. Furthermore, between SEM observation and stack dismantling, the membranes went through several manipulations (thickness and conductivity measurements, drying, SEM coating) where the weakly-bonded scaling potentially detached from the membrane, explaining the visible difference between SEM and macroscopic photographs taken just after ED treatment. However, the observations correlate with the above-mentioned results and the drastic decrease in membrane scaling, which can be attributed to the application of an appropriate combination of PEF.

## 3. Materials and Methods

### 3.1. Whey

The raw acid whey samples were obtained from Parmalat-Canada (Victoriaville, QC, Canada) processing plant by refrigerated transport at 4 °C, and then stored at −30 °C. For each run, 2 L of whey were thawed by free convection at 4 °C prior to experiments. Table 2 describes the whey composition in comparison to the one reported in literature and from our previous study.

### 3.2. Electrodialytic Configuration

**Figure 9:**
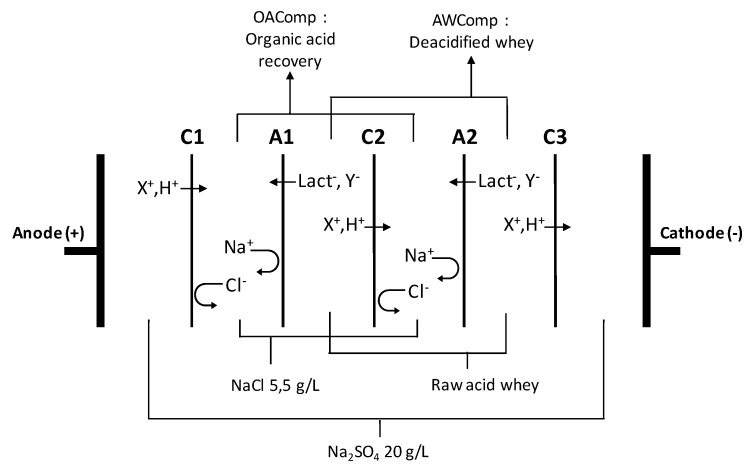
Electrodialysis (ED) configuration (CACAC, letters corresponding to the membrane’s stacking) used for acid whey deacidification. C refers to cation-exchange membrane and A to anion-exchange membrane. X^+^ and Y^−^ respectively refer to positively and negatively charged ionic species present in the whey (modified from Dufton et al. 2018 [11]).

Electrodialysis experiments were performed using an MP type cell (Multi-Purpose cell from ElectroCell AB, Täby, Sweden) with an effective surface area of 100 cm^2^. The configuration used (Figure 9) was selected due to its common use in the dairy industry and in recent studies on acid whey deacidification [10,11]. Two deacidification units were set up as to follow the previous studies, and the anode was a dimensionally stable electrode (DSA-O_2_) while the cathode was a stainless steel electrode. The potential difference was generated by a power supply (Model HPD 30-10, Xantrex, Burnaby, BC, Canada), the solutions circulated using centrifugal pumps (Baldor Electric Co., Fort Smith, AR, USA), and the flow rates controlled by flowmeters (Aalborg Instruments and Controls, Inc., Orangeburg, SC, USA).

The configuration consisted of stacking five commercial food grade cation- and anion-exchange membranes (respectively CEM and AEM). Diluate (acid whey compartment: AWComp) and concentrate (organic acid recovery compartment: OAComp) solutions were circulated between the membranes defining three closed loops. The solutions used were a 20 g/L Na_2_SO_4_ electrolyte solution (volume of 2 L, flow rate of 4 L/min), a 5.5 g/L NaCl aqueous concentrate solution (2 L, 4 L/min), and acid whey (2 L, 4 L/min). To ensure a continuous recirculation, external tanks containing the solutions were connected to each closed loop. The closest cation-exchange membrane (CEM) to the cathode (C3, see Figure 9) was added in order to avoid any anion migration in the electrolyte solution compartment, specifically lactate.

### 3.3. Protocol

In order to apply a similar driving force as in previous studies, a constant current density of 100 A/m^2^ was applied. This current density was selected as 80% of the limiting current density after its determination according to Cowan & Brown method [31]. In addition to the experiments conducted in DC current as control, two different pulse/pause combinations were tested: 25 s/25 s (ratio pulse duration/pause duration *r* = 1) and 50 s/10 s (*r* = 5) using a Pulsewave^TM^ 760 Switcher (Bio-Rad Laboratories, Richmond, CA, USA). These pulse/pause combinations were chosen with relatively long periods and high ratios, in order to allow the solution flow to rinse the membrane’s surfaces and remove the already-formed scaling, since it has been found to be plaster-like, crumbly, and easily removable by hand [11]. Such long period combinations were found to be efficient for scaling or fouling mitigation in several studies [15,16]. All experiments were conducted with different durations, according to the pulse/pause combination, or not, to correspond to an effective treatment of three hours, or to a number of charges transported of 10800 C: 3, 3.5, and 6 h for DC, 50 s/10 s and 25 s/25 s current conditions respectively. The solution tanks were kept at room temperature around 20 °C, and three replicates were performed for each current condition. At the end of each run, before dismantling the cell, the whole system (tanks, tubing, and ED cell) was rinsed for 5 min with water to remove all superficial or non-adsorbed scaling.

The raw acid whey composition was analyzed in terms of minerals, protein, lactic acid, and lactose contents before and after ED. During the ED process, the electrical conductivity and pH values were recorded every 10 minutes for each solution as well as the applied voltage. Samples were taken in both OAComp and AWComp at 0, 1800, 3600, 7200, and 10800 C (corresponding to 0, 30, 60, 120 and 180 min in DC current) for organic acid concentration determination by high-performance liquid chromatography (HPLC). Membrane thickness and electrical conductivity were measured before and after each run to ascertain membrane scaling, and the membranes were dried and kept for mineral analyses and microscopy observation.

### 3.4. Analyses

All analyses were performed on at least three technical samples.

***Total solids and ash contents***. According to the AOAC methods 990.20 and 945.46, raw acid whey samples were weighed before drying for one hour on a heating plate (Corning PC-420 Hot Plate Stirrer, NY, USA). The dried samples were then weighted again for total solid content determination, and further ashed in a furnace at 550 °C overnight until they turned white. The samples were weighed after cooling, and the ash content was determined as follows (m refers to the measured weights):

(1) $$ash,\;\%=\frac{100\times \left(m_{crucible+ashes}-m_{crucible}\right)}{m_{crucible+sample}-m_{crucible}}.$$

***Mineral composition***. Calcium, potassium, magnesium, sodium, and phosphorus concentrations were determined by optical emission spectrometry with inductively coupled plasma as atomization and excitation source (ICP-OES Agilent 5110 SVDV Agilent Technologies, Victoria, Australia), using the following wavelengths (in nm): 393.366, 396.847, 422.673 (Ca); 766.491 (K); 279.553, 280.270, 285.213 (Mg); 588.995, 589.592 (Na); 177.434, 178.222, 213.618, 214.914 (P). The analyses for all ions were carried out in axial and/or radial view, directly on acid whey samples diluted 20 times. Samples of 10 mL were diluted 1:5 in distilled water and used for ion determination.

***Organic acid contents***. Organic acid concentrations were determined by high-performance liquid chromatography (HPLC) using a chromatograph from Waters (Waters Corp., Milford, MA, USA), equipped with a Hitachi (Foster City, CA, USA) differential refractometer detector L-7490. An ICSep ICE-ION-300 column (Transgenomic, Omaha, NE, USA) was used with 8.5 mM of H_2_SO_4_ (180 µL H_2_SO_4_/L) as the mobile phase and at a flow rate of 0.4 mL/min. The column temperature was kept constant at 40 °C. Samples were centrifuged for five minutes at 5000 rpm (Allegra™ 25R Centrifuge, Beckman Coulter, Brea, CA, USA) and filtered (0.22 µm nylon; CHROMSPEC Syringe Filter, Brockville, ON, Canada) before injection (15 µL). A mixture of lactose anhydrous (PHR1025), citric acid (251275), DL-lactic acid (L1750) and acetic acid (338826) (from Sigma-Aldrich, St. Louis, MO, USA) was used as an external standard to perform the quantification in mg/L.

***Total protein content***. Total protein content was determined by the measurement of the total nitrogen concentration. This was conducted according to the Dumas combustion method using a TRUSPEC LECO FP-528 (LECO, St. Joseph, MI, USA) calibrated with EDTA. The raw acid whey was analyzed in triplicate before and after ED. A conversion factor of 6.38, proposed by Hammarsten and Sebelien in 1892, was used to determine the protein content.

***pH***. The pH of acid whey (AWComp) and organic acid recovery (OAComp) solutions were measured using a pH-meter model SP20 (VWR Symphony, Thermo Orion West Chester, PA, USA).

***Conductivity***. A YSI conductivity meter (Model 3100, Yellow Springs Instrument, Yellow Springs, OH, USA) equipped with an immersion probe (Model 3252, cell constant K = 1 /cm) was used for measuring values in acid whey (AWComp) and organic acid recovery (OAComp) solutions.

***Global system resistance***. The global system resistance (R, in Ω) was calculated according to Ohm’s law (R = U/I). The voltage (U, in V) and current intensity (I, in A) values were directly obtained from the power supply.

***Relative energy consumption (REC).***

(2) $$REC=\frac{\int_{t\;=\;0}^{t\;=\;end}\frac{U\;\times \;I}{3600}dt}{m_{lact.acid}},$$

where REC is the relative energy consumption (in Wh/g of lactic acid recovered), U the voltage applied (in V), I the applied current (in A), and $m_{lact.\;acid}$ the total mass of lactic acid recovered at the end of treatment in the OAComp (in g). The time taken into account here for the PEF conditions will be the effective time during the pulse periods.

***Membrane electrical conductivity***. Membrane conductance (G_m_) was measured using a conductivity clip (Laboratoire des Matériaux Échangeurs d′Ions, Créteil, France) with a 1 cm distance between the electrodes and the conductivity meter (Model 35, Yellow Springs Instrument Co., Yellow Springs, OH, USA). Membrane electrical resistance was calculated using the same method as Lteif et al. (1999) [32] and Lebrun et al. (2003) [33]:

(3) $$R_{m}=\frac{1}{G_{m}}=\frac{1}{G_{m+s}}-\frac{1}{G_{s}}=R_{m+s}-R_{s},$$

where R_m_ is the transverse electric resistance of the membrane (Ω), G_m_ is the membrane conductance (S), G_m+s_ is the conductance of the membrane and the solution measured together (S), G_s_ is the solution conductance (S), R_m+s_ is the resistance of both membrane and solution measured together (Ω), and R_s_ is the solution resistance (Ω). The membrane electrical conductivity was then calculated using the following relation [32]:

(4) $$K=\frac{L}{R_{m}\times A}$$

where *K* is the membrane conductivity (mS/cm), *L* is the membrane thickness (cm), *R_m_* the transversal resistance of the membrane (Ω), and *A* the electrode surface (1 cm^2^).

***Membrane mineral composition***. The same elemental concentrations as for the liquid solutions were measured for the membranes by ICP-OES, as described previously. For each configuration, the analysis was conducted in triplicate on 18.75 cm^2^ pieces of AEM (C**A**CAC the bold character highlights the membrane analyzed) and CEM (CA**C**AC). Pristine AEMs and CEMs were also analyzed as controls. Membranes pieces of 18.75 cm^2^ were cut, weighed, and dried at 60 °C overnight in an oven (VWR Gravity Convection Oven, Radnor, PA, USA). The dried samples were then ashed in a furnace at 550 °C overnight until they turned white. The samples were weighed after cooling and the ash content was determined according to Equation (1). The ashes were resolubilized in 1 mL 25% nitric acid and diluted in 50 mL total volume with demineralized water (PURELAB® Ultra, ELGA, High Wycombe, UK). The solutions were then filtered with membranes of 0.45 µm pore size before the ICP-OES analysis.

***Scanning electron microscopy (SEM) and Energy Dispersive X-ray spectroscopy (EDS) analysis***. The dried samples (same protocol as for membrane mineral composition before carbonization) were coated with a thin layer of gold to improve the image quality (Technics Hummer II Sputter Coater, Anatech Ltd., Hayward, CA, USA). Images were then registered using a field emission gun scanning electron microscope with a magnification of 50× (JMS840A SEM, JEOL, Peabody, MA, USA). The microscope was equipped with a spectrometer using the energy dispersive X-ray spectroscopy (EDS, Bruker Analysis, Billerica, MA, USA) at a 15 kV accelerating voltage and a 15 mm working distance.

***X-ray diffraction (XRD)***. The analysis was performed using a D5000 Siemens diffractometer (Montreal, QC, Canada). The radiation source (CuKα) was a copper lamp with a wavelength of 0.154 nm. The K_α_ radiation of copper was generated at 30 mA and 40 kV. The scan rate of 0.02° 2θ was applied to record patterns for 2θ ranging between 15° and 65°. Results were analyzed using JADE software version 2.1 with JCPDS database from the ICDD (International Centre for Diffraction Data) version 2001.

***Statistical analyses***. Analyses of variance (ANOVA) were performed on data and Tukey tests (α = 0.05 as probability level) were used to compare treatments (SigmaPlot software, version 12.0 for Windows, MilliporeSigma, Burlington, MA, USA).

## 4. Conclusions

There are very few reported applications of PEF on complex food matrices during ED treatments, and this was the first time that such a current mode was used on acid whey to both demineralize and deacidify it. In the present study, two pulse/pause combinations were tested: 50 s/10 s and 25 s/25 s, and were compared with DC current. Regarding migration enhancement, the PEF 25 s/25 s current condition showed a lactate migration or deacidification 16% higher than the DC condition. Moreover, supplementary analysis on whey solution showed the migration enhancement also applied to mineral ions such as calcium, magnesium, and potassium, with removal rates of 53%, 32%, and 85%, respectively, 4% to 25% higher than the rates obtained with DC current. In addition to better deacidification and demineralization efficiencies, the application of PEF conditions appeared to have a significant effect on scaling mitigation. Indeed, membrane observations and characterization allowed to correlate the drastic reduction of calcium phosphate scaling intensity with the use of pulse/pause combination of 25 s/25 s. This could also be confirmed by the 32% major reduction in final global system resistance observed in comparison with the DC condition (Supplementary Data B), as well as the 33% decrease in relative energy consumption in term of Wh per gram of lactic acid recovered. These positive effects of the 25 s/25 s PEF condition were mainly due to the reduction of concentration polarization on the membranes’ boundary layers. By decreasing such a phenomenon, (1) water splitting occurrence responsible for the local pH variations leading to scaling formation [16,21,25] was mitigated, and (2) ionic availability for migration through the membranes was improved, explaining the enhanced deacidification/demineralization rates [18,19,20]. However, it appeared crucial to pay particular attention to the pulse/pause combination applied since, in the present study, the two different PEF current conditions led to very different results regarding demineralization, deacidification, and scaling. Finally, this was the first time that PEF was demonstrated to have a simultaneous positive impact on these main concerns of ED application to complex matrices.

## Figures and Tables

**Table 1:** Total protein content in whey in g/L, before and after electrodialysis.

Time	DC	PEF 50 s/10 s	PEF 25 s/25 s
Before ED	9.70 ± 2.22a	7.56 ± 2.00a	7.35 ± 1.02a
After ED	7.67 ± 0.84a	6.35 ± 1.72a	6.84 ± 1.58a

Column marked with the same letter indicate no significant difference between the values (P > 0.05).

**Table 2:** Raw acid whey composition and physicochemical characteristics.

Composition	Unit	Acid Whey	Acid Whey from First Study [11]	Values Reported in the Literature [3,9,10,30]
Total solids	g/L	57.2 ± 1.5	59.8 ± 4.2	50.0–70.0
Total protein	g/L	7.5 ± 1.1	6.5 ± 0.7	4.2–10.0
Lactose	g/L	34.9 ± 1.0	41.2 ± 0.9	38–49
Minerals	g/L	6.9 ± 0.1	5.1 ± 1.1	4.7–7.0
P	g/L	0.76 ± 0.02	0.55 ± 0.01	0.44–0.90
Ca	g/L	1.08 ± 0.02	0.86 ± 0.02	0.43–1.60
K	g/L	1.65 ± 0.03	1.26 ± 0.05	1.28–1.82
Mg	g/L	0.10 ± 0.00	0.09 ± 0.00	0.09–0.19
Na	g/L	0.53 ± 0.02	0.39 ± 0.03	0.40–0.61
Lactate	g/L	7.12 ± 0.11	7.00 ± 0.14	5.18–8.00
Ratio Lactate/Lactose	No unit	0.20	0.17	0.12–0.15
pH	No unit	4.6	4.4	4.0–4.6
Conductivity	mS/cm^2^	7.09 ± 0.35	7.05 ± 0.24	8.27 ± 0.42

## Supplementary Materials

Supplementary materials can be found at http://www.mdpi.com/1422-0067/20/4/797/s1.
