# Supplementary material for: Positive Impact of Pulsed Electric Field on Lactic Acid Removal, Demineralization and Membrane Scaling during Acid Whey Electrodialysis
Source: Int J Mol Sci. 2019 Feb 13;20(4):797. doi: 10.3390/ijms20040797 (PMC6412636; doi:10.3390/ijms20040797)
Supplement: Supplementary file 1 [file ijms-20-00797-s001.pdf]

Supplementary data

Supplementary data A.

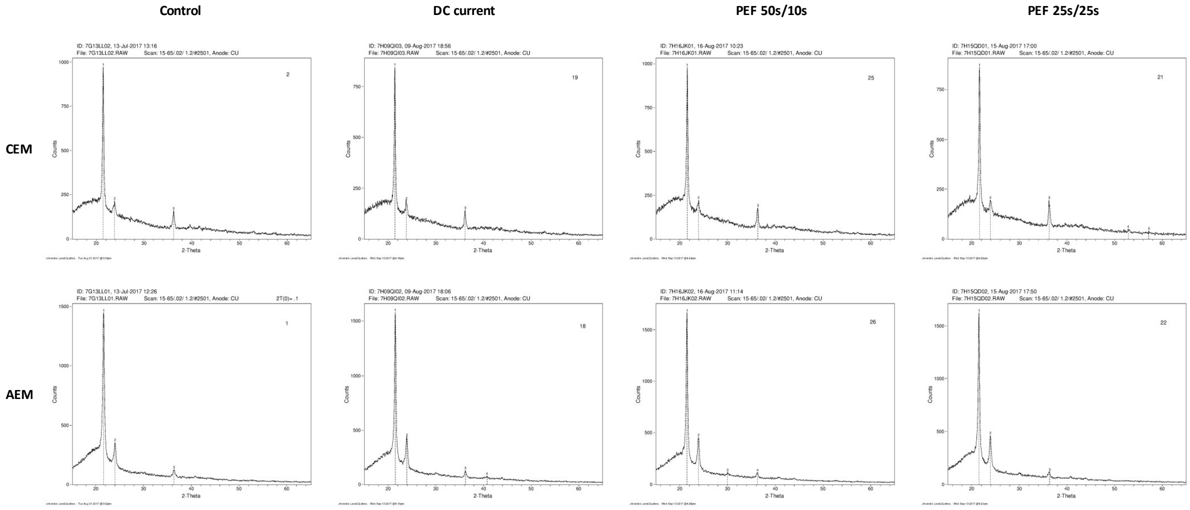

Figure 1: x-Ray diffraction of anion (AEM) and cation exchange membranes (CEM) for all current conditions. Both sides of each membrane were analyzed and gave similar results.

Supplementary data B.

Table 1: Removal rates of calcium, potassium and magnesium in the whey after ED.

	**Ca**	**K**	**Mg**
		Unit : %	
**DC current**	45.00 ± 1.67a*	81.27 ± 0.27a	25.95 ± 2.80a
**50s/10s**	45.67 ± 1.31a	80.90 ± 0.13a	26.77 ± 1.62ab
**25s/25s**	52.84 ± 1.15b	85.14 ± 0.41b	32.45 ± 1.84b

** Using the same letter means that there is no significant difference between the values (P>0.05).*
